# Editorial

**DOI:** 10.1080/10647440300025501

**Published:** 2003

**Authors:** Sebastian Faro


*Infect Dis Obstet Gynecol 2003;11:73*

### Editorial

The European Society of Infectious Diseases in Obstetrics and Gynecology has selected our journal, Infectious Diseases in Obstetrics and Gynecology, as the official publication of their society. Now three societies, IDSOG, I-IDSOG-USA, and ESIDOG, have chosen our journal as their official publication. I anticipate that our journal will serve as a forum for both investigators and clinicians to raise questions about data contained in published articles. I also hope it will provide a mechanism for interested individuals to raise questions regarding both treatment recommendations and protocols presented for the management of various infections. Therefore, this journal will not only disseminate results of current investigations, but also stimulate discussions and ideas regarding a variety of infections unique to the obstetric and gynecologic patient.

The editors take this opportunity to welcome our colleagues from Europe. We also encourage our readers to become more active in the journal by submitting letters to the editor concerning the articles we publish, infectious diseases affecting women, and new developments in the areas of antimicrobials, probiotic treatments, vaccines, and diagnostic tests. The journal should also be a forum for individuals to express their opinions regarding infectious diseases based on scientific evidence.

Recently we have seen an increase in the number of manuscripts received. The quality of submissions also continues to improve. However, our journal is still faced with the task of increasing the number of subscriptions. It is anticipated that with three societies selecting our journal as their official publication, new subscriptions will follow. It is incumbent on all members of these societies to submit quality manuscripts and encourage their associates to subscribe to the journal.

Sebastian Faro, MD, PhD
Editor-In-Chief

